# Editorial: Microbial comparative genomics and pangenomics: new tools, approaches and insights into gene and genome evolution

**DOI:** 10.3389/fgene.2024.1490645

**Published:** 2024-10-24

**Authors:** Digvijay Verma, Tulasi Satyanarayana, Paulo Jorge Dias

**Affiliations:** ^1^ Department of Environmental Microbiology, School of Earth and Environmental Sciences, Babasaheb Bhimrao Ambedkar University, Lucknow, Uttar Pradesh, India; ^2^ Department of Biological Sciences and Engineering, Netaji Subhas University of Technology, New Delhi, India; ^3^ iBB - Institute for Bioengineering and Biosciences, Instituto Superior Técnico, University of Lisbon, Lisbon, Portugal; ^4^ Associate Laboratory i4HB - Institute for Health and Bioeconomy at Instituto Superior Técnico, University of Lisbon, Lisbon, Portugal

**Keywords:** pangenomics, microbial genomics, next-generation sequencing, whole genome sequencing, metagenome

Developments in Genomics and DNA Sequencing technologies complement each other. The genesis of Comparative Genomics evolved during the late 70s and early 80s, when the first generation of efficient DNA sequencing technologies had been developed by Walter Gilbert and Frederick Sanger (Shendure et al., 2017). Subsequently, more reliable, and automated sequencing machines were developed by Applied Biosystems and their dissemination to research laboratories in the 90s that led to a major leap in Comparative Genomics (Shendure et al., 2017). Thus, the focus of researchers gradually shifted from small-scale DNA analysis (e.g., genes) to whole genome sequence analysis, an era inaugurated with the release of first complete genome of a free-living organism, the bacterium *Haemophilus influenzae* (Fleischmann et al., 1995), followed by the first sequenced genome of a eukaryotic organism, *Saccharomyces cerevisiae* (Goffeau et al., 1996), culminating in the first draft genome sequence of *Homo sapiens* (Lander et al., 2001; Venter et al., 2001).

The emergence of Next-Generation Sequencing (NGS) technologies was the game changer during the first decade of the 21st century that led to the development of a new sub-field of Genomics, i.e., Pangenomics. The initial concept of Pangenome fulfilled the demand for minimum inclusion of the required number of genomes to achieve good confidence by covering the entire genes of a species (Tettelin et al., 2005). The entire spectrum of genes, gene order, gene content, and their structural variations could be explored to assess the species’ phylogenetic relatedness. This gene-oriented branch of genomics categorizes genes into three groups based on their relatedness among various strains of a species. This includes core (shared among 99% of the strains), shell (10–99%), and, cloud (<10% of the strains) genes. The core genes are evolutionarily conserved, while shell and cloud genes constitute variable regions. Microbial Pangenomics grabbed attention in 2005 when researchers compared whole genome sequences of eight strains of *Streptococcus agalactiae* that unveiled several new genes into the assembly (Tettelin et al., 2005). Palsson’s team explored pangenomic analysis of several strains of *Escherichia coli* (Monk et al., 2013) and *Mycobacterium tuberculosis* (Kavvas et al., 2018) and their comparison to identify closely as well as distantly related genes. The second decade of this century witnessed the birth of the 3rd generation of sequencing technologies. This led to the evolution of the concept of Pangenomics. With this new concept, Pangenomics is now viewed as an advanced approach to Genomics that considers the entire genomic diversity of a species or organisms, where every base matters. This also gave rise to a new discipline of Computational Pangenomics, responsible for the development of efficient data structures, algorithms, and statistical methods to perform bioinformatic analyses on computational objects known as Pangenome Graphs.

This Research Topic highlights the duality that exists between Genomics and DNA Sequencing technologies. Many of the sixteen articles presented here used sequencing approaches to explore various facets of Pangenomics and Comparative Genomics. The following text provides a summary of exciting studies from the experts in this area.

Whole genome sequencing (WGS) analysis performed by Zhang et al. on three different isolates of *Streptococcus equi* originating from donkeys showed a high degree of resemblance to each other. A comparative analysis of these strains with a genome sequence of a horse isolate *S. equi* 4047 unveiled several rearrangements and inversions in their genomes. Prophage and other virulence factors were identified to determine their genomic diversity and virulence factors. A similar finding was recorded by Shikov et al., where the group observed host specificity among the members of *Serratia marcescens*. Prophages were specific for humans, while genetic islands were specific for plants and insects. A comparative genomics of Malonate Semialdehyde Decarboxylases (*MSAD*) gene from *Mycobacterium* spp. performed by Lee et al. revealed the absence of MSAD-like genes (*MSAD-1* and *MSAD-2*) in the pathogenic species of *Mycobacterium*. Thus, MSAD loss suggested host-specific adaptations among pathogenic mycobacterial species. In another investigation on *Mycobacterium* genomes, Mei et al. report the isolation of *Mycobacterium tuberculosis* (MTB) strains from patients with Cutaneous Tuberculosis (CTB) and the corresponding genomic characterization. The study concludes that these isolates predominantly belong to the Beijing lineage, consistent with the present epidemic status of the MTB disease in China. Besides, different infection sites of MTB in patients are independent of any association with specific genomic changes. *M. tuberculosis* pangenome was also constructed revealing the lack of significant differences in the accessory genome among different types of strains associated with the CTB disease.

Comparative genomics offers to unveil the alteration in the genomes that occurs due to various adaptations. A comparative pangenome analysis of 371 different genomes of *Arcobactor* species conducted by Zhou et al. successfully identified a taxonomic marker gene (*gene* 711) for effective classification of *Arcobactor* spp. over the ANI (Average Nucleotide Identity) and isDDH (*in silico* DNA–DNA hybridization) like traditional methods of bacterial identification. Yang et al. present a new bioinformatics pipeline based on the PanGenome Graph Builder (PGGB), allowing the semi-automated construction of a Pangenome Graph. The resulting computational object represents the genomic relationships among the highly recombinant genome sequences of *Neisseria meningitidis* strains, showing enhanced detection capability of genetic variations. This pipeline allows the identification of new virulence and antimicrobial resistance genes to facilitate vaccine design and quick detection of bacterial outbreaks, with an impact on public health surveillance. The pangenomics-based variant analysis, population genetics, and evolutionary biology can be studied effectively. Pangenome analysis of *Akkermansia* (the only known genus of Verrucomicrobiota) performed by Dámariz González et al. revealed that the variation in its genome is due to horizontal gene transfer either from unknown species or from Gram-negative gut bacteria. Moreover, the analysis revealed that genes involved in mucin degradation, surface interaction, and, adhesion are constituents of the last *Akkermansia* common ancestor (LAkkCA). The mucin degradation ability of *Akkermansia* is an essential feature of being a symbiotic species. The study conducted by Morales-Olavarría et al. on pangenomics concluded that *Porphyromonas gingivalis* and *Porphyromonas gulae* maintain a strong core-to-accessory ratio for housekeeping genes, and the differences in their genomes are chiefly due to involvement of genes of unknown functions. Esteves et al. developed a binary matrix (0, 1) based protocol to develop effective biomarkers for identifying amorphic sequence mutations. The technique can be employed on other MRSA clones or from other bacterial species. This technique overcomes the problems associated with the WGS and the requirement of trained personnel. Confirming the increasing importance of Artificial Intelligence (AI) algorithms in Comparative Genomics, two studies published in this topic have used deep learning (DL) algorithms to analyze two distinct types of biological problems, the prediction of protein structure and RNA modification. Besides pangenomics, Li et al. developed a proteome analysis using a deep learning (DL) algorithm to study the *Acidithiobacillus* genome (a well-known bacterium involved in bioleaching process for metal recovery from ores). DL assisted in gene ontology in terms of 93.6% unknown proteins. To date, the crystal structures of only 14 distinct proteins of this bacterium are known, further studies are welcome in this line of work. Yu et al. have taken advantage of autoBioSeqpy, a Keras-based deep learning software for fast and easy development, training, and analysis of deep learning model architectures for biological sequence classification, to develop a DL model able to predict the 5-methyluridine (m5U) modification. The authors concluded that the model combining convolutional neural network (CNN) and bidirectional long short-term memory (BiLSTM) was consistently better than all other DL models tested, being named Deepm5U. This model showed a predictive performance that outperformed the current state-of-the-art tool.

Zhao et al. use long-read sequencing based on the latest generation of Oxford Nanopore Technology (ONT) to identify MultiDrug Resistance (MDR) genes in *Salmonella* strains. The authors focus their attention on the spread of MDR-associated genes carried out by plasmids since these mobile genetic elements can be transferred easily between different bacterial species, enabling a quick and effective dissemination of such genes, and causing important public health issues. WGS was used by Villacís et al. in the identification of the pathogen *Raoultella ornithinolytica*. These authors propose the replacement of the automated identification methodology based on phenotypic characteristics with this new methodology to ensure accuracy. This study also reports an evolutionary origin of certain MDR genes that are relatively recent in this human pathogen, a fact consistent with the open Pangenome attributed to *R. ornithinolytica* and with the worrying trend of quick acquisition and dissemination of antibiotic resistance genes observed in this *Enterobacteriaceae* species. WGS was also explored by Tokuda et al. to analyze phyllody-inducing genes (also known as phyllogens) in various *Phytoplassma* species and strains. The authors showed that the flanking sequences of these genes act as Potential Mobile Units (PMUs), facilitating the horizontal transfer of phyllogens. This study also showed that phyllogen function and the corresponding coding sequences are highly conserved, a fact suggesting that these genes are essential for survival of their *Phytoplasma* hosts. Finally, in this Research Topic, two research groups have analyzed important human pathogens and the corresponding epidemic status in different regions of China. In the first study, Zhang et al. used NGS to determine the sequence of 16S rDNA gene of the whole microbiome of parasitic ticks in Wuwei City, showing the existence of a wide range of bacterial species and high phylogenetic diversity in the corresponding microbiomes. A network analysis of the detected bacterial genera showed that depending on the genera, microbial combinations might be promotive or inhibitive of co-infection. In another study, Zhang et al. analyzed the most common human pathogen responsible for gastrointestinal diseases, *Helicobacter pylori*, in Ningbo, China. These authors used multi-locus sequence typing to show that this bacterial species has a high prevalence of mixed infections, having identified 246 new alleles and 53 new sequence types. This study also confirmed a high genomic diversity present in this human pathogen in this country and showed the prevalence of mixed infections in Ningbo was higher than the ones reported in other regions of China.

Looking ahead, the awesome ability of Artificial Intelligence in the recognition of patterns, and the anticipated dissemination of these methodologies to all areas of scientific knowledge combined with the advent of the first commercial generation of quantum computers at the turn of the decade and the corresponding leap in the computational power available to biologists anticipate a new major leap in Comparative Genomics and Pangenomics. The development of new methodologies combining these technological revolutions suggests that soon biologists will be able to directly simulate the phenotype of microbial strains and species from the observed genotypes, leading to the evolution of the current descriptive nature of Comparative Genomics and Pangenomics towards a new one more predictive. The future thus looks bright.
